# New perspectives on migraine treatment: a review of the mechanisms and effects of complementary and alternative therapies

**DOI:** 10.3389/fneur.2024.1372509

**Published:** 2024-05-09

**Authors:** Xiaoli Song, Qian Zhu, Lanqian Su, Lei Shi, Hao Chi, Yalan Yan, Mei Luo, Xibin Xu, Baohong Liu, Zhengyang Liu, Jin Yang

**Affiliations:** ^1^First Teaching Hospital of Tianjin University of Traditional Chinese Medicine, Tianjin, China; ^2^National Clinical Research Center for Chinese Medicine Acupuncture and Moxibustion, Tianjin, China; ^3^Clinical Medical College, Southwest Medical University, Luzhou, China; ^4^Tianjin University of Traditional Chinese Medicine, Tianjin, China; ^5^Tianjin Gong An Hospital, Tianjin, China; ^6^Evidence Based Oriental Medicine clinic, Sioux Falls, SD, United States

**Keywords:** migraine, pain management, alternative therapy, acupuncture, complementary therapy

## Abstract

Migraine is a prevalent and disabling neurovascular disorder, with women being more susceptible, characterized by unilateral throbbing headache, often accompanied by nausea and vomiting, and often associated with various comorbidities such as brain and cardiovascular diseases, which can have a serious impact on quality of life. Although nonsteroidal anti-inflammatory drugs (NSAIDs) are the main first-line medications for the treatment of pain, long-term use often leads to side effects and drug addiction, which emphasizes the need to investigate alternative pain management strategies with fewer adverse effects. Complementary and alternative medicine is a viable pain intervention often used in conjunction with traditional medications, including acupuncture, herbs, moxibustion, transcutaneous electrical stimulation, bio-supplements, and acupressure, which offer non-pharmacological alternatives that are now viable pain management options. This review focuses on the mechanistic doctrine of migraine generation and the role and potential mechanisms of Complementary and Alternative Therapies (CAT) in the treatment of migraine, summarizes the research evidences for CAT as an adjunct or alternative to conventional therapies for migraine, and focuses on the potential of novel migraine therapies (calcitonin gene-related peptide (CGRP) antagonists and pituitary adenylyl cyclase-activating peptide (PACAP) antagonists) with the aim of evaluating CAT therapies as adjunctive or alternative therapies to conventional migraine treatment, thereby providing a broader perspective on migraine management and the design of treatment programs for more effective pain management.

## 1 Introduction

Migraine is a recurrent neurovascular disorder clinically characterized by unilateral throbbing moderate to severe headaches, often accompanied by other symptoms such as nausea and vomiting, and sensitivity to light and sound (1). According to epidemiologic studies, the incidence is 12–15% in the general population, and women are more commonly affected than men, especially in the most fertile age group, 25 to 55 years (2). Migraine, as the second most disabling neurological disorder, has co-morbid relationships with a variety of brain disorders (e.g., cerebral infarction, cerebral hemorrhage), cardiovascular disease, and epilepsy, and is a significant cause of disability (3). Migraine arises from a series of intracranial and extracranial changes due to neuronal dysfunction and carries the risk of changing from episodic migraine to chronic migraine, especially as the frequency of attacks increases and acute care medications are overused (4, 5).

Traditional treatments for migraine include a variety of acute care options (e.g., over-the-counter pain relievers (sometimes in combination with caffeine), nonsteroidal anti-inflammatory drugs, opioids) and migraine-specific medications (e.g., tretinoin and ergot) (6). Recent advances include the approval of CGRP antagonists for migraine prophylaxis in adults, such as erenumab, fremanezumab, and galcanezumab (7). While these therapeutic agents are effective in many individuals, they may not be appropriate for all patients, and some have contraindications or potential side effects (6). In addition, overuse of acute medications can lead to chronicity of migraine (8, 9).

Complementary and alternative therapies (CAT) are being explored as potential alternative treatments. They are becoming more widely recognized as a viable option for pain management because of their ability to relieve stressful effects, reduce recurrence and prevent chronic pain (10). CATs encompass a variety of forms including, but not limited to, transcutaneous electrical stimulation, herbs, acupuncture, acupressure, moxibustion, qigong, tai chi, yoga, and meditation (10, 11). These therapies include non-pharmacological options such as electrical nerve stimulation devices and magnetic stimulation devices that target various nerves such as the trigeminal, vagus and occipital nerves (12). Behavioral medicine techniques, such as biofeedback training and positive thinking, have also been used for some time to help manage migraines (13). These alternative therapies can provide more options for patients seeking relief from migraine symptoms, especially those who have not responded well to traditional therapies or are looking for non-pharmacological treatments. Research indicates that in the treatment of migraine, these alternative therapies demonstrate significant advantages that cannot be overlooked. For instance, acupuncture, a common form of CAT, not only matches the efficacy of mainstream pharmacological treatments but also offers a lower risk of side effects, providing patients with a safer and more appealing treatment option (14). Consequently, as our understanding of CAT deepens, these therapies not only offer a diverse array of treatment options for migraine sufferers but also drive innovation in chronic pain management, contributing to enhanced treatment outcomes and improved quality of life for patients.

## 2 Mechanisms of action of migraine

### 2.1 Migraine mechanisms in neurobiological and physiological perspective

**Figure 1:**
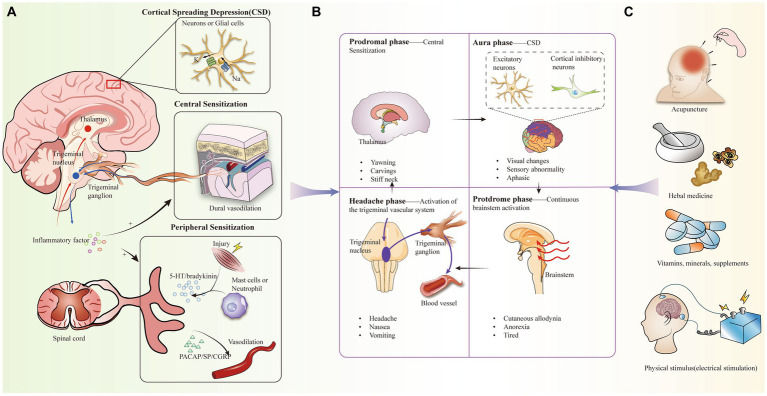
**(A)** Pathogenesis of migraine, including activation of the trigeminal vascular system, central and peripheral sensitization, cortical spreading inhibition, inflammation. **(B)** Clinical manifestations of the four periods of migraine and the corresponding mechanistic doctrines. **(C)** Complementary alternative therapies for migraine headaches.

Despite the fact that the pathogenesis of migraine is not clearly understood, there have been several theories that attempt to explain its cause, including vascular dysfunction, aseptic inflammatory response in the dura mater, and magnesium deficiency (15–17) (Figure 1A). Not only that, but there is evidence to support that migraine with aura is associated with cortical spreading depression (CSD), in which depolarizing waves generated by neurons and glial cell membranes in the cerebral cortex diffuse themselves along the cortex, leading to activation of trigeminal afferent pathways (18, 19). In particular, the caudal subnucleus of the spinal trigeminal nucleus (STN) sends out nociceptive-sensitive nerve fibers that transmit information about perceptual stimuli to the thalamus, leading to sensitization of tertiary neurons. During the diffusion of signals from the cerebral cortex, CSD may be associated with large potassium (K^+^) efflux, sodium (Na^+^) voltage-sensitive channel opening, and glutamate release (20).

In addition, most scholars now believe that activation of the trigeminal vascular system (TGVS) better explains the cause of migraine. Migraine attacks begin with triggers, especially migraine-inducing factors that alter central excitability, such as stress, sleep deprivation, fasting, and sound (21). Under these stimuli, the trigeminal nervous system is sensitized, which in turn induces the trigeminal ganglion (TG) to release a variety of neuropeptides, including CGRP, substance P (SP), and pituitary adenylate cyclase-activating polypeptide (PACAP) to participate in the neuroinflammatory response. At the same time, as TGVS is in a chronically activated state, it leads to a series of other changes, including mast cell degranulation and changes in meningeal vasodilatation. More interestingly, CSD can alter the permeability of the blood–brain barrier through activation and upregulation of matrix metalloproteinases (22, 23).

Considering that CGRP, as a key peptide, plays an important role in pain signaling, it has been demonstrated that CGRP release can be inhibited using herbs (24). The transient receptor potential ankyrin (TRPA) mediates CGRP release in neurogenic inflammation, and the study by Benemei et al. (25) demonstrates that Petasin inhibits CGRP signaling, achieving this through desensitization of TRPA. New studies have recently found that the PACAP pathway is independent of the CGRP pathway, and there are findings suggesting that frequent headache-induced reductions in PACAP and subsequent up-regulation of PACAP receptors play an important role in migraine progression (26–28). Therefore, PACAP antagonists may be a new therapeutic option for patients who are insensitive to CGRP antagonists (28). Perhaps, in the therapeutic regimen for migraine, CAT may play a role by inhibiting the PACAP signaling pathway. In addition, when treating chronic migraine and hyperparathyroidism (PTH), it may be more effective to consider combined inhibition of the CGRP and PACAP signaling pathways rather than inhibition of a single one of these signaling pathways (29).

### 2.2 Inflammation and migraine

Epidemiologic studies have found that 2.5–3% of patients with episodic migraine (EM) transition to chronic migraine (CM) in the second year (21, 30). The mechanism of its chronicity may be closely related to peripheral sensitization of primary afferent nerve fibers, secondary neurons in the STN, and central sensitization of higher neurons such as the thalamus (31, 32). In addition, the inflammatory response is closely related to peripheral sensitization and increased central sensitivity (33, 34). When activation of injury receptors occurs, primary afferent neurons, mast cells, and eosinophils in local tissues release a variety of chemicals, such as 5-hydroxytryptamine (5-HT) and bradykinin, which promotes neuroinflammation and modulates pain (35, 36). Among them, 5-HT 1F receptor agonists (e.g., Lasmiditan) are already in clinical trials (37). In addition, the introduction of CGRP as a target has been an important advance in migraine medication (38). CGRP levels increase when migraine attacks occur and decrease after treatment, thereby attenuating the vasodilating potency and central sensitizing effects of the pro-inflammatory neuropeptide CGRP, which has been confirmed in numerous studies to be a key neurotransmitter involved in migraine attacks (39, 40). A range of CGRP receptor antagonists and monoclonal antibodies to CGRP are currently in clinical trials, opening up new possibilities for migraine treatment drugs (41).

## 3 Applications and research evidences of cat in migraine management

Migraine is categorized into a prodromal symptomatic phase, an aura phase, a headache phase, and a late headache phase, with headache phase symptoms manifesting as recurrent pain, nausea, and vomiting (32, 42–44). Currently, tretinoin, a 5-hydroxytryptamine 5-HT1B/1D receptor agonist, is the migraine-specific acute treatment of migraine during the headache phase, but it is not suitable for every patient, while the prophylactic effect is not good, and in recent years, the more promising alternative for acute-phase treatment has been complementary alternative therapies (CAT) (45) (Figure 1B). CAT has been used in the treatment of a wide range of pains, including mind–body interventional therapies (e.g., meditation), biologic based therapies (e.g., taking herbs and vitamins, dietary supplementation), physical therapy, and manual therapies including acupuncture (41, 46) (Figure 1C). Despite some methodological challenges, the effectiveness of these CAT modalities is supported by several studies (47) (Table 1).

**Table 1:** Clinical evidence for CAT in the treatment of migraine.

Statistical methods	Types of CAT	Research and intervention groups	Interventions	Results	Conclusions	Limitations	Reference
Multicenter, Randomized, Controlled, Blinded	Acupuncture	150 Acupuncture Primary Treatment of Migraine Patients with Episodic Migraine without Aura	20 sessions of complementary acupuncture treatment	Patients who underwent acupuncture therapy in the experimental group had a significant reduction in the number of migraine attacks at weeks 13–20 and a significant reduction in the frequency of migraine attacks at weeks 17–20	Preventing migraine attacks without aura with 20 treatments of hand acupuncture is superior to sham acupuncture and usual care	The lack of baseline prophylaxis is not typical; the time frame of the study was not long enough	(48)
Randomized, Controlled	Acupuncture	48 participants: 10 controls and 38 migraineurs	Two sessions of 5 days each, 1 day between sessions (11 days total)	VAS, PSQI, and MSQ were medically statistically significant in patients treated with acupuncture	Acupuncture is effective in relieving migraine symptoms	The psychological assessment scale lacked assessment of pain status	(49)
Double-blind, Randomized, Controlled	REN	Sixty-five migraine patients underwent multifocal rTMS	Dot-burst stimulation at 67 Hz, 85% RMT, and 8 s column-to-column spacing	Migraine patients treated with real rTMS had a lower average number of migraine days per month; the rate of reduction in migraine attack frequency was higher	Multifocal rTMS is an effective and well-tolerated prophylactic treatment for episodic migraine patients	5 patients withdrew, with missing data and loss to visit bias	(50)
Randomized, Controlled	REN	CM patients (18–55 years old) with International Classification of Headache Disorders, Third Edition (ICHD-3) β-criteria	10 Hz rTMS applied with a figure-of-eight magnetic stimulation coil three times a month, one day apart, for three months	More than 50% reduction in the number of headache days and 50% reduction in headache severity at 3 months in group II compared with group I	rTMS combined with amitriptyline is safer and more effective in treating CM than rTMS alone	Approximately 50% of patients in group I are transferred to group II due to inadequate headache relief	(48)
Prospective controlled clinical trial	Massage Therapy	16 female patients with migraine	Eight female migraineurs underwent 12 sessions of CTM for four weeks.	Significant changes in pain, concomitant symptoms (except vomiting), medication use, Headache Impact Test-6, and Disability with Migraine Disability Assessment Scale (DMDAS) scores in the CTM group compared with the control group	CTM can be considered a non-pharmacologic and complementary therapy for migraine	Only female patients were tested, there was selection bias; the sample size was too small, the reproducibility and representativeness of the study results were poor, and false-negative or false-positive conclusions may be drawn	(70)
Randomized, Controlled	Reflexology	48 women (33–58 years old) with migraine for 2–10 years admitted from November 2013 to November 2015	The RG group received two 10-treatment sessions per week; the SMG group received three 15-treatment sessions per week.	All variables (VAS, IA, FA, DA) within the RG and SMG were reduced from baseline values at 3 months after treatment	Reflexology and segmental massage offer a safe alternative to pharmacologic treatment of migraine. Migraineurs derive significant health benefits from foot reflexology	Short follow-up period; small sample size	(71)
Double-blind, Placebo-controlled	Ginger	107 patients (18–60 years old) with episodic migraine, not taking any prophylactic medications	3 capsules of 200 mg of dried ginger extract (5% active ingredient) or placebo (cellulose) each time	The number of days of severe pain, analgesic use for acute migraine, and duration of migraine attacks were reduced in both groups, but there were no significant differences between groups	Ginger has no greater benefit in the prophylactic treatment of migraine compared to placebo	High placebo response; lack of pharmacologic evaluation of ginger capsules	(51)
Randomized, Single-center, Double-blind, Parallel, Controlled	Magnesium	260 migraineurs (18–65 years old) one month of no prophylaxis, 3 months of therapy	Randomized to 3 intervention groups receiving oral sodium valproate tablets, magnesium sodium valproate tablets, and magnesium oxide tablets twice daily for 12 weeks	All migraine characteristics were significantly reduced in all three groups compared with those reported at baseline; MIDAS and HIT-6 scores were significantly lower in Groups A, B, and C, and these changes were more pronounced in Groups A and B than in Group C	Magnesium enhances the antimigraine properties of valproate in combination therapy and reduces the dose of valproate required for migraine prophylaxis	Some participants did not participate in blood collection; lack of complete data on serum magnesium levels limited the analysis of the correlation between serum magnesium levels and treatment efficacy in the three groups of patients	(54)
Cross-sectional	Magnesium	3,626 participants (20–50 years old) in the 2001–2004 National Health and Nutrition Examination Survey (NHANES)	Dietary magnesium intake determined by 24-h retrospective method and supplemental magnesium intake determined by dietary supplement interviews	Mean dietary magnesium intake was below the RDA in both migraine and control groups; In the adjusted model, dietary and total magnesium intake were associated with lower odds of migraine in the lowest Q	Inadequate Magnesium Intake Linked to Migraine in U.S. Adults 20–50 Years of Age	Cannot explain temporal relationship between magnesium ingestion and migraine; residual confounding after adjusting for modeling; single question for assessing migraine	(52)
Randomized, Multicenter, Double-blind, Placebo-controlled	Magnesium, CoQ10 and Riboflavin	130 adult migraineurs (18-65 years) with ≥ 3 migraine attacks per month	2 capsules of a proprietary supplement containing magnesium, riboflavin and coenzyme Q10 were taken orally in the morning and evening for 3 months	Migraine frequency decreased and intensity was significantly lower in the supplement group	Treatment with supplements containing magnesium, riboflavin, and coenzyme Q10 reduced migraine frequency; the Migraine symptoms and disease burden significantly reduced in dietary supplementation group	Unblinding patients in the verum group due to chromaturia	(56)

REN, Remote Electrical Nerve Stimulation; VAS, visual analogue scale; PSQI, Pittsburgh sleep quality index; MSQ, MinnesotaSatisfaction Questionnaire; rTMS, Repetitive Transcranial Magnetic Stimulation; CTM, connective tissue massage; IA, intensity of attacks; FA, frequency of attacks; DA, duration of attacks; MIDAS, Migraine Disability Assessment; HIT-6, Headache Impact Test-6; RDA, recommended dietary allowance; RG, reflexology group; SMG, segmental massage group.

Migraine may be associated with electrolyte disturbances, and magnesium deficiency may induce migraine by affecting cortical inhibition or leading to abnormalities in glutamatergic neurotransmission, which is seen as a potential mechanism for the magnesium-migraine association (53). Magnesium is involved in the regulation of the nervous system through multiple pathways, not only regulating vasodilatation by affecting mitochondrial metabolism, neurotransmitter release, and substance P release, but also attenuating neuroinflammation by inhibiting the nuclear factor κB pathway in pro-inflammatory cells (54, 55). Given the close link between inflammation and migraines, employing magnesium as a supplement emerges as a potent approach for the mitigation or prophylaxis of migraine episodes. In CAT, there have been several randomized clinical controlled trials supporting the use of magnesium as a supplement for the prevention of migraine attacks; however, most of the studies have been combination treatments in conjunction with other vitamins or bioorganic molecules (56). Using a randomized, multicenter, double-blind controlled trial, Gaul et al. demonstrated that treatment with supplements containing magnesium, riboflavin, and coenzyme Q10 reduces the frequency of migraine attacks, their clinical symptoms, and the burden of disease (56). Among these, riboflavin may protect nerves by reducing inflammation and anti-oxidative stress properties, suggesting its potential as a migraine preventive agent (57).

Herbal treatment has the advantages of holistic conditioning, multi-targeting, and long-lasting effects, which are conducive to individualized and fine-tuned treatment for migraine patients (58–60). In 22 years, Yang et al. (61) showed that in a rat model of nitroglycerin (NTG)-induced migraine, the Chinese herbal formula Xiongshao Zhitongfang (XZR) regulated NO, 5-HT, CGRP, and SP to normal levels, while inhibiting mast cell degranulation and the release of inflammatory factors, which resulted in attenuation of migraine symptoms. In addition, in a homozygous rat model, rhubarb extract from the traditional Chinese medicine *Rheum palmatum* also down-regulated the inflammatory response and alleviated migraine via the cGMP-PKG pathway (62). However, there is not much research available on how herbs can holistically condition the body and its long-term effects in treating migraines.

In addition to the commonly used pharmacological complementary alternative therapies, non-pharmacological treatments such as Remote Electrical Nerve Stimulation (REN), massage therapy, due to fewer adverse events, then it may be a more promising mode of treatment for migraine (63, 64). In randomized controlled trials, the frequency of migraine attacks was reduced in patients treated with rTMS therapy; also, rTMS was safer and more effective in treating chronic migraine (CM) when combined with amitriptyline (50, 65). The mechanisms involved may be related to the regulation of central and peripheral sensitization by rTMS (66).

From the perspective of qi and yin and yang concepts of Chinese medicine, acupuncture is based on meridians placing needles or pressing on specific locations on the patient’s skin to achieve therapeutic effects; from the perspective of physiology, the stimulation of high-threshold and tiny nerve fibers can transmit signals to specific brain regions mirrored by acupuncture points, leading to the release of endogenous opioids to achieve analgesic effects (67). Functional magnetic resonance imaging (MRI) data support that areas of brain activity in migraine patients who undergo acupuncture include the limbic system and the default mode network, as well as pain processing areas. The increased ALFF (Amplitude of Low-Frequency Fluctuation) values in these areas suggest that acupuncture may enhance spontaneous brain activity in patients with migraines (49). Acupuncture has been shown to be superior to sham surgery and placebo (68, 69). Meta-analyses have been performed to show that acupuncture reduces the frequency of migraine attacks more than pharmacologic prophylaxis and is less likely to result in withdrawals and reports of adverse effects due to adverse reactions (68).

## 4 Limitations of research on the use of cat for migraine treatment

Despite the large number of randomized controlled trials showing the benefits of complementary alternative therapies for migraine treatment, most studies have limitations, focusing mainly on methodological challenges (47). Common reasons for this include short follow-up time, small sample size, and patient loss, which leads to poor reproducibility and representativeness of the study results (70, 71). The limited availability of diagnostic criteria is also a challenge. Current diagnostic criteria do not adequately capture the heterogeneity of migraine, including underlying genetic and neurobiological factors. For example, a controlled trial of acupuncture treatment was unable to hypothesize a link between psychological and pain states because the Psychological Assessment Scale lacked an assessment of pain states (49). In addition, a recent meta-analysis showed a trend toward higher placebo responses in migraine prevention trials over the last 30 years (72). Another promblem is the impact of differences in patterns of regional culture. In Asian cultures, herbal treatments and tai chi are widely popular. However, these non-mainstream medical approaches may limit acceptance and application in non-Asian populations (73, 74).

## 5 Discussion

Migraine is a mechanistically complex disorder caused primarily by neurovascular disorders, and its pathologic and physiologic processes are evolutionary and do not consist of a single mechanistic doctrine (75). Genetic and epigenetic susceptibility may also explain the development of migraine (76–78). Large genome-wide association studies have shown that genetics may contribute to altered brain morphology in individuals at high risk for migraine. Although genome-wide association studies have identified many susceptibility variants, including genetic factors shared with comorbidities, more in-depth studies exploring the overall susceptibility loci for migraine are needed to understand the cellular phenotypes resulting from migraine gene variants (26).

The design of future studies of complementary alternative treatments for migraine should ensure methodological rigor, reproducibility, and safety. 0Incorporation of CAT into migraine treatment should take into account the frequency of visits, the patient’s expectations of the treatment, and the psychological response to the treatment setting in order to avoid a high placebo response (26). Although most treatments are well tolerated with limited adverse effects, the possible risk of death due to carotid artery entrapment with high-speed chiropractic and hepatotoxicity of pyrrolizidine alkaloids in butterbur cannot be ignored (47, 79, 80). In addition, key areas of migraine research include further exploration of molecular markers and the use of imaging techniques to identify key mechanisms and triggers. In a longitudinal neuroimaging study, the duration of the aura phase of a spontaneous human migraine attack was found to be 48 h using MRI, and hypothalamic activation may serve as a potential marker for this staging (75, 81). In summary, the potential of CAT in migraine treatment is remarkable, offering a range of pharmacological and non-pharmacological options that can be tailored to the therapeutic needs of individual patients. While current research supports the efficacy of various CAT modalities, it is clear that more rigorous studies are needed to fully understand the mechanisms and optimize their integration into clinical practice.

## Author contributions

XS: Conceptualization, Writing – original draft, Writing – review & editing. QZ: Data curation, Writing – original draft. LaS: Conceptualization, Writing – original draft. LeS: Writing – original draft. HC: Data curation, Writing – original draft. YY: Writing – original draft. ML: Methodology, Writing – original draft. XX: Writing – original draft. BL: Data curation, Writing – original draft. ZL: Data curation, Writing – original draft. JY: Data curation, Funding acquisition, Writing – original draft, Writing – review & editing.
